# Causes and Management of Halitosis: A Narrative Review

**DOI:** 10.7759/cureus.43742

**Published:** 2023-08-19

**Authors:** Rita M Khounganian, Osama N Alasmari, Mohammed M Aldosari, Nawaf M Alghanemi

**Affiliations:** 1 Dentistry, College of Dentistry, King Saud University, Riyadh, SAU

**Keywords:** probiotics, causes, mouth wash, treatment, management, halitosis

## Abstract

Halitosis is defined as the presence of an unpleasant odor in exhaled air, regardless of its cause. In most patients with halitosis, the condition causes embarrassment and interferes with social interactions and daily life. Furthermore, bad breath can be a sign of an underlying disease. Understanding the factors and causes that lead to halitosis and its manifestations could facilitate proper management of this condition. To properly diagnose and treat patients, healthcare professionals, including primary care physicians and dental professionals, must be familiar with the etiology and appropriate management of the disease. Consequently, this review aims to provide practitioners with up-to-date information on the etiological factors of halitosis to facilitate the establishment of preventive measures and provide accurate diagnosis and management.

## Introduction and background

The American Dental Association defines halitosis as a combination of the Latin and Greek words halitus (breath) and osis (pathological process) [1]. Halitosis is a condition that causes an unpleasant odor in the exhaled air [1]. Halitosis is categorized into genuine halitosis, pseudo-halitosis, and halitophobia [2]. Genuine halitosis is a socially unacceptable form of malodor [2]. Pseudo-halitosis is a condition in which the patient complains of having bad breath; however, others are unable to detect it clinically through scientific testing [3]. Patients still believe that they have bad breath even though the problem has been completely treated and there is no clinical or social evidence of their complaints. This condition is referred to as halitophobia [4].

Because some individuals are unaware of their bad breathing conditions, the prevalence of halitosis may be underestimated [5]. A study conducted by Al-Ansari et al. in 2006 on a population sample of 1500 people showed that 25% of the population had halitosis [6]. In addition, Liu et al. conducted a study on 2000 individuals in China, and 25% of this population demonstrated halitosis [7].

Halitosis can affect an individual’s life, and its psychological effects can lead to social anxiety disorders that decrease social communication [8,9]. Halitosis can affect productivity, and most patients with halitosis complain of personal discomfort [10]. This review aims to provide practitioners with up-to-date information on the etiological factors of halitosis to provide preventive measures and the most effective treatment.

## Review

Methodology

This review article involved an assessment of published studies discussing the management and causes of halitosis. Multiple databases, including PubMed, Web of Science, and Google Scholar, were utilized to collect the most relevant articles on this subject. The search used several terms, such as halitosis, halitosis treatment, and halitosis causes. By using this method, all the studies discussing halitosis causes, management, and manifestations were obtained. We included all studies that addressed halitosis and its causes, management, and manifestations. Studies with poor methodological quality, outdated data, or insufficient data were excluded. The initial screening elicited 158 studies. After implementing our inclusion criteria, the most relevant articles were selected and included in this review. This review ultimately involved 57 papers related to halitosis causes and management.

Causes of halitosis

Murata et al. classified halitosis into extra-oral halitosis (EOH) and intraoral halitosis (IOH) [11]. More than 85% of halitosis cases are due to intraoral conditions, such as tongue coating and poor oral hygiene [12]. According to Silveira et al., halitosis is caused by the synthesis of volatile sulfur compounds (VSCs) by bacteria [13]. Gram-negative bacteria that contribute to IOH exhibit a high load in the posterior region of the tongue [14]. This is primarily due to the "cratered" tongue surface, consisting of a complex papillary structure that facilitates the retention of large quantities of bacteria such as Fusobacterium nucleatum and Streptococcus spp. The presence of these two bacterial strains in individuals with IOH exhibited a significant association [15,16]. Fixed orthodontic appliances, large caries, or any other factor that increases the chance of food and plaque accumulation and facilitates bacterial retention can cause IOH due to the ability of bacteria to produce VSCs [17,18]. A dry mouth may be associated with halitosis, which could be explained by the fact that diminished salivary flow promotes anaerobic bacterial putrefaction of food debris left in the oral cavity after eating [19].

The EOH accounts for 5%-10% of all halitosis cases [17]. Moreover, ear-nose-throat diseases account for 10% of halitosis cases, whereas 5% of individuals have halitosis due to gastrointestinal disorders [12]. Several conditions can cause halitosis, including diabetes, liver disease, and kidney disease [20]. There are also physiological causes such as dehydration, starvation, dry mouth, advanced age, and some types of food [17]. Smoking is another cause of halitosis. Several studies have found a correlation between smoking and a higher risk and severity of halitosis [21].

Manifestations of halitosis

Halitosis has various negative effects on life, including social, occupational, and emotional restrictions that reduce self-assurance, spontaneity, and self-worth [22]. A recent study conducted on Chinese patients revealed that those with halitosis had worse psychological status than those without halitosis. However, this negative effect was not related to the severity of halitosis [23]. According to a recent systematic review, adolescents and young adults who experienced halitosis felt more anxious and depressed [24].

It has been suggested that oral health can affect quality of life [25,26]. An observational study concluded that Brazilian adolescents who self-reported halitosis were strongly associated with a higher impact on quality of life [27]. A recent systematic review also concluded that halitosis negatively impacts oral health-related quality of life [28].

Prevention of halitosis

Enhancing individuals' understanding of halitosis and its etiology can aid in its prevention [29]. Additionally, providing oral hygiene instructions and implementing measures to ensure patient adherence to self-care practices would substantially contribute to the prevention of this condition [29]. Avoiding a diet high in volatile foods, such as onions, garlic, and spices, can aid in the prevention of halitosis [30]. Breakfast consumption has been linked to a lower risk of halitosis [31]. Furthermore, avoiding alcohol and tobacco use is also recommended because of the negative effects these habits have on bad breath [32]. Also, by consistently practicing oral hygiene through frequent toothbrushing, flossing, and the use of mouth rinses, the likelihood of experiencing bad breath is reduced [32].

Diagnosis of halitosis

A thorough oral examination is required as the initial step in the diagnosis of halitosis in order to determine the local underlying factors and causes [33]. Halitosis can be assessed using both instrumental and organoleptic methods [33]. Organoleptic measurement is the most popular diagnostic procedure [34,35]. In the organoleptic approach, the air that is emitted from the mouth and nose is smelled to determine whether or not it has an unpleasant smell [36]. The malodor strength is then graded on a scale of 0 to 5, with 0 denoting a lack of malodor and 5 denoting a very significant malodor [36]. For instrumental measurement, various devices are used, including gas chromatographs, electronic noses, and sulfide monitors [37]. By determining the concentration of VSCs, gas chromatography is utilized in halitosis studies to assess breath odor [34]. While costly and impractical for daily usage, this approach is extremely sensitive and specific [34]. While less expensive and less bulky than traditional gas chromatographs, electronic portable instruments for gas analysis, like the Halimeter (a portable sulfide monitor) (Interscan, Chatsworth, USA), are less versatile in their capacity to evaluate various gases [34].

Management of halitosis

Elimination of etiologic factors and improvement in oral health status are the cornerstones of halitosis management [38]. Halitosis can be a symptom of an underlying systemic disease; therefore, an accurate diagnosis of its origin (intraoral or extraoral) is essential for proper management [39]. Periodontal disease and caries, both of which contribute to halitosis, should be addressed to eliminate these conditions [38,40]. Good oral hygiene is an essential factor in the treatment of halitosis [38,40]. Treatment methods include the chemical and mechanical reduction of microorganisms, odor-masking products, and chemical neutralization of VSCs [1,40,41]. Recently, Renvert et al. stated that primary care physicians, dental professionals, and psychiatrists should work on a team-based approach to treat halitosis [12].

Mechanical scrubbing of the dorsum of the tongue is a viable treatment for halitosis [42]. Van der Sleen et al. concluded that mechanical scrubbing of the dorsum of the tongue has major implications in reducing tongue coating, which is the foremost cause of halitosis [42]. The use of mechanical scrubbing with or without chemical substances reduces the bacterial load, thereby aiding in the elimination of the condition [43]. The tongue can be scraped with a variety of tools, and by applying slight pressure, most of the tongue coating can be removed [44]. A clinical trial conducted on periodontitis-free, non-smoking participants who practiced routine oral hygiene by brushing or scraping their teeth for two weeks established a negligible decrease in the number of germs on their tongue and, sequentially, a considerable decrease in the tongue coating. Consequently, tongue cleaning appears to reduce substrate decomposition rather than heavy bacterial loads [45]. In a randomized clinical trial conducted in 2019, Gurpinar et al. concluded that patients who were assigned to the tongue scraper group showed a lower Halimeter score than the mouthwash group. They had been instructed to use a tongue scraper to clean the dorsum of their tongues. Before and after using the tongue scraper, subjects were instructed to thoroughly cleanse it with clean water. The individuals were instructed to use the brush in gentle circular strokes for two minutes, starting as far back along their tongues as possible and moving forward [46].

The pharmacological inhibition of VSC-producing bacteria is an effective way to treat halitosis [41]. Several over-the-counter mouth rinses inhibit VSC-producing bacteria [41]. Lower organoleptic scores were achieved using mouth rinses containing amine fluoride, stannous fluoride, zinc, chlorhexidine, or cetylpyridinium chloride [41]. In a clinical investigation conducted in 2000, volunteers were exposed to 3% hydrogen peroxide, and researchers discovered a 90% reduction in all three VSCs [47]. A meta-analysis and systematic review conducted in 2022 revealed that chlordioxides are strong oxidizing agents that can reduce halitosis by oxidizing hydrogen sulfide, methyl mercaptan, cysteine, and methionine [48]. A 29% reduction in odor was noted after four hours [48]. Moreover, full-mouth disinfection procedures play a significant role in diminishing VSCs, consequently leading to a reduction in halitosis [13]. The implementation of full mouth disinfections involves the use of scaling and root planing procedures within 24 hours, in combination with the application of chlorhexidine in all intraoral pockets, followed by chlorhexidine mouthwash for no more than fifteen days [13,49].

Probiotics are a relatively new treatment for halitosis. They are living microorganisms that, when administered in sufficient amounts, improve host health [50]. A probiotic gum containing Lactobacillus reuteri was assessed for its effect on halitosis in 25 healthy young adults (mean age: 22 years) who self-reported malodorous morning breaths. Organoleptic ratings improved after probiotic gum use; however, no change in the quantity of VSC was found to account for this improvement [51]. The effect of a probiotic tablet containing Lactobacillus salivarius and xylitol (280 mg) on halitosis was studied in a double-blind, crossover, randomized, placebo-controlled clinical trial. The trial was completed by 23 patients (19 women and four men; aged 22-67 years) [52]. In the first crossover phase, the test group consumed the probiotic tablet for 14 days, whereas the placebo group consumed only the placebo tablet. The groups were switched to the second crossover phase (14 days) following a two-week washout period. During the clinical trial, one tablet was administered orally three times a day after meals and oral cleaning. In both periods, the organoleptic test scores were substantially lower than their respective baseline scores (P < 0.001 and P = 0.002, respectively). Moreover, no differences were observed between the periods. This study also revealed that the concentration of VSCs decreased considerably (P = 0.019) during the probiotic period compared to the placebo period.

Despite encouraging results from the previously mentioned randomized controlled trials, a recent systematic review concluded that there is no convincing evidence to support the use of probiotics in the treatment of halitosis [53]. Future research using probiotics as an intervention requires standardized techniques for the recruitment of individuals with halitosis and organoleptic assessments [53].

Antimicrobial photodynamic therapy is a medical intervention that employs the combination of light and photosensitive compounds to target and destroy the cell wall of bacteria, causing cell death [54,55]. The primary etiological cause of halitosis is anaerobic bacteria. Consequently, this therapeutic approach has been successful in terms of reducing VSC and decreasing the bacterial load on the dorsum of the tongue [54,56]. This method has numerous advantages, such as the minimization of tissue injury and the prevention of bacterial resistance development [54,56]. According to recent systematic reviews, antimicrobial photodynamic therapy has the ability to efficiently eliminate bacteria responsible for the production of VSC, and it is an effective approach for managing halitosis for a longer duration compared to conventional treatments such as tongue scrapers for this condition [54,57].

## Conclusions

Halitosis can negatively affect the quality of life. The most important aspects of halitosis management are accurate diagnosis and identification of the etiology. A multidisciplinary approach is necessary for prevention, accurate diagnosis, and management. Because halitosis is a relatively common condition, dental practitioners should be aware of its management.
